# Epithelial–Mesenchymal Transition: A Major Pathogenic Driver in Idiopathic Pulmonary Fibrosis?

**DOI:** 10.3390/medicina56110608

**Published:** 2020-11-13

**Authors:** Francesco Salton, Barbara Ruaro, Paola Confalonieri, Marco Confalonieri

**Affiliations:** Pulmonology Department, University Hospital of Cattinara, 34149 Trieste, Italy; barbara.ruaro@yahoo.it (B.R.); paola.confalonieri.24@gmail.com (P.C.); marco.confalonieri@asugi.sanita.fvg.it (M.C.)

## Editorial

Idiopathic pulmonary fibrosis (IPF) is a progressive disease of the lungs that leads to parenchymal scarring and death due to respiratory failure within a few years despite the recent therapeutic advances [1]. Diagnostic criteria rely on a combination of radiological and histopathological features, which can be simplified into identification of the usual interstitial pneumonia (UIP) pattern and exclusion of alternative diagnoses [2].

Two main aspects are noteworthy in a critical view of IPF: first, truly idiopathic diseases do not exist in biology, but they are classified as such due to an incomplete understanding of their underlying mechanisms; second, fibrosis is a physiological process that follows repair after injury when complete regeneration is not achievable, thus it only turns into disease when it becomes disproportionate and/or progressive. Indeed, it is not the UIP pattern itself that distinguishes IPF from other interstitial lung diseases most, but its progressive behavior.

The currently prevailing hypothesis for IPF pathogenesis holds that the fibrotic cascade starts from defective alveolar epithelial type-II (ATII) cells, which are not able to trans-differentiate into the more widespread alveolar epithelial type-I (ATI) cells and consequently to regenerate the alveolar epithelium like they normally do after injury or during turnover.

We have reviewed some of the dramatic progresses that have been recently made in the understanding of the pathogenic mechanisms of IPF, with special regard to the role of epithelial–mesenchymal transition (EMT) [3].

EMT is a biological process in which, under several stimuli, epithelial cells lose their normal polarity and shape to acquire mesenchymal features of migration and extracellular matrix production [3]. Despite the fact that EMT is a common downstream mechanism of all fibrosing diseases, whether it represents a leading process in the development of IPF has been widely discussed and is still a matter of debate.

One milestone on this topic is the lineage-tracing study by Rock et al., who found that only a relatively small number of fibroblasts derive from epithelial cells and that myofibroblast markers (α-smooth muscle actin) do not co-localize with EMT-derived cells in the bleomycin-induced mouse model of lung fibrosis. The authors conclude that no contribution to the expansion of mesenchymal population comes from EMT in this experimental setting [4].

On the contrary, Chilosi and coauthors gave the first demonstration that, in human tissue samples of idiopathic pulmonary fibrosis, known markers of major EMT signaling pathways (ZEB1 and β-catenin) are concurrently expressed both in fibroblast foci and in damaged epithelial cells, providing indirect evidence that EMT plays a role in human pathology. While this finding contrasts what was previously observed in vivo with the robust technique of lineage tracing, it must be stressed that the bleomycin-induced mouse model of IPF is possibly a suboptimal one, as fibrosis develops in response to acute inflammatory damage and tends to be self-resolving, contrary to what happens in human disease [5].

Subsequent studies reported that platelet-derived growth factor (PDGF), transforming growth factor beta-1 (TGFβ1), tumor necrosis factor (TNF) and other mediators that play a role in the migration, proliferation and activation of fibroblasts, are expressed by hyperactivated lung epithelial cells. Furthermore, TGFβ1 has been shown to interact with developmental pathways that are overexpressed in IPF epithelial cells (e.g., Wnt and Sonic Hedgehog), converging to EMT via the activation of SMAD3 [6] and recent findings confirmed that ATII cells undergoing EMT promote, through paracrine signaling, a microenvironment that activates local fibroblasts, enhancing the pro-fibrotic drive in a loop departing from the epithelium [7].

The sum of this evidence supports the new interpretative key for IPF pathogenesis that has emerged in recent years: ATII stemness failure, as a consequence of the ATII-to-ATI trans-differentiation blockade, seems to be responsible for the hyperactivation of ATII cells and the upregulation of several upstream pathways that are major drivers of EMT, besides directly stimulating fibroblast proliferation and collagen secretion in a paracrine fashion [8].

In other words, according to this hypothesis, the abnormal occurrence of EMT contributing to lung fibrosis is thought to be indirectly stimulated by an impairment in the process that physiologically allows for the regeneration of the alveolar epithelium, which could be properly named “epithelial-to-epithelial transition (EET)”.

Important knowledge was added to the field after the publication of our review paper. Notably, Su et al. found that RAS-responsive element binding protein 1 (RREB1) is a key partner of TGF-β-activated SMAD transcription factors in EMT [9], while Qian and coauthors identified the oncogene metastasis-associated protein 1 (MTA1) to be significantly upregulated in TGF-β-mediated EMT both in vitro and in vivo [10]. Recently, Saito et al. demonstrated that the constitutionally activated mammalian target of Rapamycin (mTOR) pathway in the ATII cells of transgenic mice resulted in an exacerbation of bleomycin-induced lung fibrosis and that mTOR-transfected ATIIs showed an enhanced expression of EMT markers after treatment with TGFβ1 [11]. Lastly, Wu et al. stated that mechanical stretch is able to activate a TGF-β signaling loop in ATII cells, which drives the periphery-to-center progression fibrosis typical of IPF [12]. The authors did not investigate the activation of proper EMT pathways in their model of disease; however, it is likely that this occurs according to the ensemble of other findings and previous evidence [13,14]. This provides fundamental—yet indirect—support to EMT being a major player in IPF, as mechanical stretch has been shown to explain the spatial distribution of fibrosis in human pathology [15].

Given the tight relationship between the epithelium and endothelium in the alveolus, future research should also be directed towards investigating the mechanisms of endothelial–mesenchymal transition (endoMT) and, in general, the possible contribution of endothelial cells to the development of fibrosis.

It is likely that the increasing recognition of precise molecular mechanisms and factors of genetic susceptibility will soon allow for both the removal of the wording “idiopathic” from “IPF” and the identification of more effective therapeutic targets. In fact, the two commercially available drugs for IPF, Pirfenidone and Nintedanib, decelerate the progression of the disease by interfering with EMT among several other processes; however, any future therapeutic approach will likely be a game-changer only if it will be able to restore the normal epithelial function instead of just blocking the late function of fibrosis.

In conclusion, despite the fact that huge advances in the understanding of IPF are still needed to pave the way for the development of new and more effective therapies, we have re-analyzed the concept of EMT and discussed its contribution to the pathogenesis of fibrosis, supporting the most recent hypothesis, according to which it may be considered not as a primitive mechanism of disease, but rather as an alternative process to dysfunctional EET.

## References

[1] Richeldi L., Collard H.R., Jones M.G. (2017). Idiopathic pulmonary fibrosis. Lancet.

[2] Raghu G., Remy-Jardin M., Myers J.L., Richeldi L., Ryerson C.J., Lederer D.J., Behr J., Cottin V., Danoff S.K., Morell F. (2018). Diagnosis of Idiopathic Pulmonary Fibrosis. An Official ATS/ERS/JRS/ALAT Clinical Practice Guideline. Am. J. Respir. Crit. Care Med..

[3] Salton F., Volpe M.C., Confalonieri M. (2019). Epithelial-mesenchymal transition in the pathogenesis of idiopathic pulmonary fibrosis. Medicina.

[4] Rock J.R., Barkauskas C.E., Cronce M.J., Xue Y., Harris J.R., Liang J., Noble P.W., Hogan B.L.M. (2011). Multiple stromal populations contribute to pulmonary fibrosis without evidence for epithelial to mesenchymal transition. Proc. Natl. Acad. Sci. USA.

[5] Chilosi M., Caliò A., Rossi A., Gilioli E., Pedica F., Montagna L., Pedron S., Confalonieri M., Doglioni C., Ziesche R. (2017). Epithelial to mesenchymal transition-related proteins ZEB1, β-catenin, and β-tubulin-III in idiopathic pulmonary fibrosis. Mod. Pathol..

[6] Selman M., Pardo A. (2020). The leading role of epithelial cells in the pathogenesis of idiopathic pulmonary fibrosis. Cell. Signal..

[7] Hill C., Jones M.G., Davies D.E., Wang Y. (2019). Epithelial-mesenchymal transition contributes to pulmonary fibrosis via aberrant epithelial/fibroblastic cross-talk. J. Lung Health Dis..

[8] Moimas S., Salton F., Kosmider B., Ring N., Volpe M.C., Bahmed K., Braga L., Rehman M., Vodret S., Graziani M.L. (2019). miR-200 family members reduce senescence and restore idiopathic pulmonary fibrosis type II alveolar epithelial cell transdifferentiation. ERJ Open Res..

[9] Su J., Morgani S.M., David C.J., Wang Q., Er E.E., Huang Y.H., Basnet H., Zou Y., Shu W., Soni R.K. (2020). TGF-β orchestrates fibrogenic and developmental EMTs via the RAS effector RREB1. Nature.

[10] Qian W., Cai X., Qian Q., Zhang W., Tian L. (2020). Metastasis-associated protein 1 promotes epithelial-mesenchymal transition in idiopathic pulmonary fibrosis by up-regulating Snail expression. J. Cell. Mol. Med..

[11] Saito M., Mitani A., Ishimori T., Miyashita N., Isago H., Mikami Y., Noguchi S., Tarui M., Nagase T. (2020). Active mTOR in Lung Epithelium Promotes Epithelial-Mesenchymal Transition and Enhances Lung Fibrosis. Am. J. Respir. Cell Mol. Biol..

[12] Wu H., Yu Y., Huang H., Hu Y., Fu S., Wang Z., Shi M., Zhao X., Yuan J., Li J. (2020). Progressive Pulmonary Fibrosis Is Caused by Elevated Mechanical Tension on Alveolar Stem Cells. Cell.

[13] Cabrera-Benítez N.E., Parotto M., Post M., Han B., Spieth P.M., Cheng W.E., Valladares F., Villar J., Liu M., Sato M. (2012). Mechanical stress induces lung fibrosis by epithelial-mesenchymal transition. Crit. Care Med..

[14] Heise R.L., Stober V., Cheluvaraju C., Hollingsworth J.W., Garantziotis S. (2011). Mechanical stretch induces epithelial-mesenchymal transition in alveolar epithelia via hyaluronan activation of innate immunity. J. Biol. Chem..

[15] Carloni A., Poletti V., Fermo L., Bellomo N., Chilosi M. (2013). Heterogeneous distribution of mechanical stress in human lung: A mathematical approach to evaluate abnormal remodeling in IPF. J. Theor. Biol..

